# “Thinking about Not-Thinking”: Neural Correlates of Conceptual Processing during Zen Meditation

**DOI:** 10.1371/journal.pone.0003083

**Published:** 2008-09-03

**Authors:** Giuseppe Pagnoni, Milos Cekic, Ying Guo

**Affiliations:** 1 Department of Psychiatry and Behavioral Sciences, Emory University School of Medicine, Atlanta, Georgia, Untied States of America; 2 Emory University School of Medicine, Atlanta, Georgia, Untied States of America; 3 Department of Biostatistics, The Rollins School of Public Health, Emory University, Atlanta, Georgia, Untied States of America; University of Minnesota, United States of America

## Abstract

Recent neuroimaging studies have identified a set of brain regions that are metabolically active during wakeful rest and consistently deactivate in a variety the performance of demanding tasks. This “default network” has been functionally linked to the stream of thoughts occurring automatically in the absence of goal-directed activity and which constitutes an aspect of mental behavior specifically addressed by many meditative practices. Zen meditation, in particular, is traditionally associated with a mental state of full awareness but reduced conceptual content, to be attained via a disciplined regulation of attention and bodily posture. Using fMRI and a simplified meditative condition interspersed with a lexical decision task, we investigated the neural correlates of conceptual processing during meditation in regular Zen practitioners and matched control subjects. While behavioral performance did not differ between groups, Zen practitioners displayed a reduced duration of the neural response linked to conceptual processing in regions of the default network, suggesting that meditative training may foster the ability to control the automatic cascade of semantic associations triggered by a stimulus and, by extension, to voluntarily regulate the flow of spontaneous mentation.

## Introduction

There has been a resurgence of scientific interest in the neurophysiological bases of meditation in recent years [1], [2], owing in part to the wide availability and increasing sophistication of *in vivo* brain imaging techniques. An important aspect of these practices that has not been directly investigated, and the subject of the present work, is the relationship between meditation and conceptual processing. The Buddhist meditative exercise has its roots in the metaphysical tenet of “emptiness,” particularly emphasized by the Zen schools [3]. According to this view, reality is originally devoid of ontological properties and it is only via an incessant and largely unconscious habit of emotional self-reference and categorization that a conceptual structure is created and ultimately reified; a process necessary for daily life, but that also tends to condition the individual into predefined patterns of thoughts, feelings, and behaviors. Meditation is believed to counteract this tendency in favor of a condition of equanimity where the provisional nature of one's own conceptual structure is realized, bringing about a greater freedom of thought and action as well as a decreased sense of self-attachment.

The classical instructions for the practice of *zazen* (“seated meditation”) can be found in the XII century text *Fukan Zazengi* by Dōgen Kigen, the patriarch of the Japanese Sōto Zen school:

*“Think of neither good nor evil and judge not right or wrong. Stop the operation of the mind, and consciousness; bring to an end all desires, all concepts and judgments […] After the bodily position is in order, regulate your breathing. If a thought arises, take note of it and then dismiss it. When you forget all attachments steadfastly, you will naturally become zazen itself.”* [*4*] *.*

In cognitive terms, the attempt at mental regulation through meditation involves developing a progressive familiarity with the interplay of voluntary attention (often directed to the breath and/or the posture) and the spontaneous conceptual processing that appears in its fractures, a process facilitated by the adoption of a stable seated posture and a quiet environment. It should also be noted that while particular meditative practices attempt to promote absorption and sensory withdrawal from the environment (see [5], [6], for a classification of meditative techniques), Zen meditation, quite to the contrary, prescribes a vigilant attitude that is pragmatically implemented by the adoption of a seated posture with a certain degree of active tension and by keeping the eyes open; mental withdrawal from the environment is considered as promoting a state of dreaminess and lack of clarity counterproductive to the meditative pursuit and is therefore vigorously discouraged [7].

The study of spontaneous cognitive processes in the resting state has recently acquired some momentum due to the neuroimaging finding of a consistent set of brain regions displaying higher activity during wakeful rest than during a variety of demanding tasks. Such a “default mode of brain function” [8], [9] has in fact been implicated in the spontaneous stream of thoughts, episodic memories, and conceptual processing that normally occurs in the absence of goal-directed activity [10], [11], [12], [13], and which appears to be integral to our sense of self [14], [15], [16], [17]. The default network includes regions in the medial prefrontal cortex, posterior cingulate, angular gyrus, and the left superior and middle frontal gyri [8], [9], [18], [19], while typical subcortical components are the hippocampus and parahippocampal gyrus [14], [20], [21].

In the current study, we tested the hypothesis that the habitual practice of being heedful to distraction from spontaneous thoughts during meditation renders regular meditators, as compared to control subjects, more able to voluntarily contain the automatic cascade of conceptual associations triggered by semantic stimuli. To this purpose, we adapted a simple lexical decision task [11] that required the subjects to decide whether the visually presented stimuli were real English words or strings of letters with plausible readings but no semantic content (“nonwords”) by pressing a button on an MRI-compatible response device. The stimuli were delivered on a temporally sparse schedule within an ongoing meditative condition: subjects were instructed to attend to their breathing throughout the scan, perform the lexical decision task when a stimulus appeared on the screen, and promptly re-focus their attention to their breathing. We hypothesized that the default network in meditators would display a response associated with semantic processing characterized by a reduced duration compared to control subjects, for whom the cascade of conceptual associations triggered by semantic stimuli would be less effectively terminated by the experimental prescription of redirecting attention to the breathing.

## Results

### Behavioral data

A repeated-measure ANOVA with group (CTRL, MEDT) as a between-subject factor and stimulus type (word, nonword) as a within-subject factor, yielded a significant effect of stimulus type on the response times to the lexical decision task (*F*(1,22) = 13.23, *p* = 0.0015), but no effect of group (*F*(1,22) = 0.26, *p* = 0.62) and no interaction of group by stimulus type (*F*(1,22) = 2.84, *p* = 0.11). The number of omissions and errors was very small and similar across groups and stimulus types (Table 1).

**Table 1:** Behavioral data for the lexical decision task: mean response times (in ms, st. dev. in parentheses), mean number of errors, and omissions (st. dev. in parentheses).

	*words*	*nonwords*
*Response times*		
CTRL	987.9 (266.8)	1094.3 (341.6)
MEDT	969.4 (182.1)	1008.4 (208.7)
*Errors*		
CTRL	0.8 (1.2)	1.0 (1.0)
MEDT	0.9 (0.7)	0.8 (0.9)
*Omissions*		
CTRL	1.2 (1.9)	0.8 (1.5)
MEDT	1.0 (1.7)	0.8 (1.9)

### Imaging data

**Figure 1:**
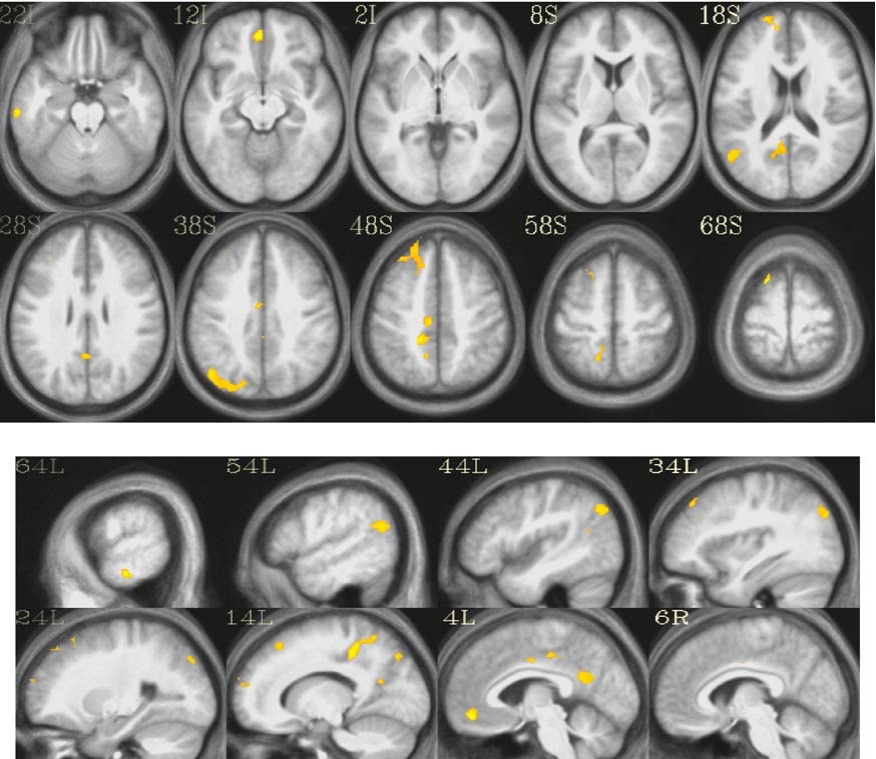
Activated clusters for the contrast *words-nonwords* on the pooled data (CTRL+MEDT). The *t*-map is thresholded at *p*<0.001, *k*>27 voxels (α<0.05).

**Figure 2:**
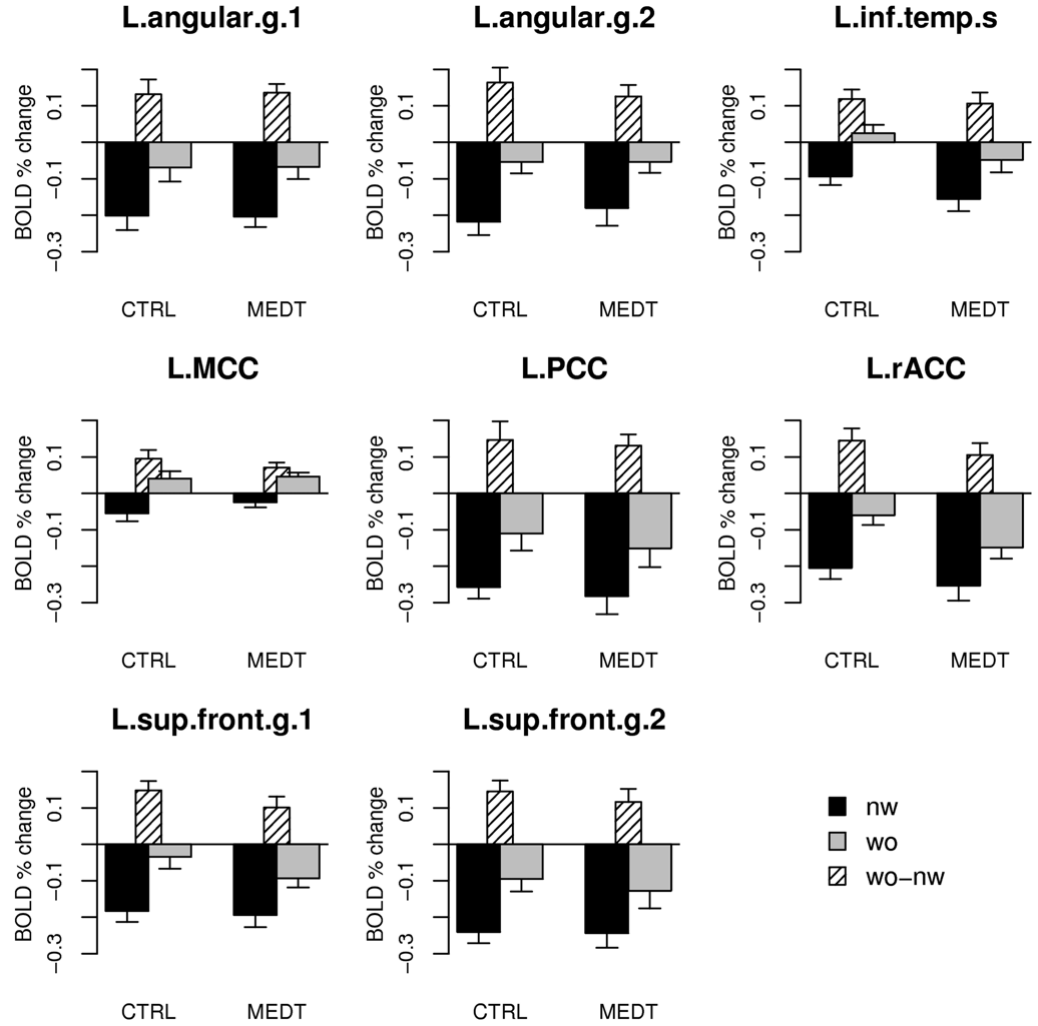
ROI-based averages of the Gamma model beta coefficients for words (“wo”) and nonwords (“nw”) in controls (CTRL) and meditators (MEDT). Abbreviations for ROI names are the same as in Table 2, where the index 1 and 2 for clusters with the same anatomical label follows the order in the table.

The contrast *words-nonwords* in the random-effects analysis on the pooled data (CTRL+MEDT) identified a collection of areas in the left hemisphere largely overlapping with the default mode network [8] (Table 2 and Figure 1). In order to examine the results in more detail within the regions detected by the pooled analysis, we extracted the ROI-based average values of the estimation coefficients (“betas”) for the word and nonword regressors (Figure 2).

**Table 2:** Clusters of activation for the contrast *words-nonwords* on the pooled data (CTRL+MEDT), thresholded at *p*<0.001 and *k*>27 voxels (α<0.05).

*Region*	*size*	*t-value*	*x*	*y*	*z*
L MCC	195	6.08	−9	−24	45
L sup frontal g (1)	146	5.68	−18	18	66
L sup frontal g (2)	44	5.20	−18	60	18
L angular g (1)	145	5.52	−45	−69	39
L angular g (2)	69	6.17	−51	−63	21
L PCC	82	5.26	−6	−54	21
L rACC	46	6.10	−3	45	−12
L inf temporal g	27	5.80	−63	−21	−21

Cluster sizes are in voxels, t-values refer to the peak voxel in the cluster, and stereotactic coordinates are in MNI space (mm). Abbreviations: L = Left, MCC = middle cingulate cortex, PCC = posterior cingulate cortex, rACC = rostral anterior cingulate cortex, g = gyrus, s = sulcus, sup = superior, inf = inferior. The indices (1) and (2) are used to distinguish different clusters in similar anatomical locations.

An aspect of the results portrayed in Figure 2 that may appear puzzling at first is that the observed response to the stimuli was generally a deactivation compared to baseline, for both words and nonwords. This is perhaps unexpected, given the amount of existing data implicating regions of the default mode network in semantic processing, especially on the left side [10], [11], [13], [22], [23], [24]. It can be explained, however, by considering that (1) some activity related to spontaneous thoughts is likely to be present during the meditative baseline condition, and (2) the response to the stimuli in our task always included the interruption of an ongoing state of introspectively oriented attention to require visual processing and a motor response (see Methods). While this task-switching component is likely to be responsible for the general deactivation induced by both words and nonwords [25], it was important to verify that the observed differential activity induced by words and nonwords in regions of the default mode network was not due to a simple difference in processing difficulty [26], as suggested by slower reaction times for nonwords compared to words (Table 1), rather than to semantic processing. We therefore performed a Pearson correlation analysis across subjects between the values of the *words-nonwords* contrast in each ROI and the average difference in response times between word and nonword stimuli. All correlations were non-significant, even when omitting the correction for multiple testing, with very low values for the correlation coefficient (all *p*>0.05, uncorrected; median *r* = 0.04).

**Figure 3:**
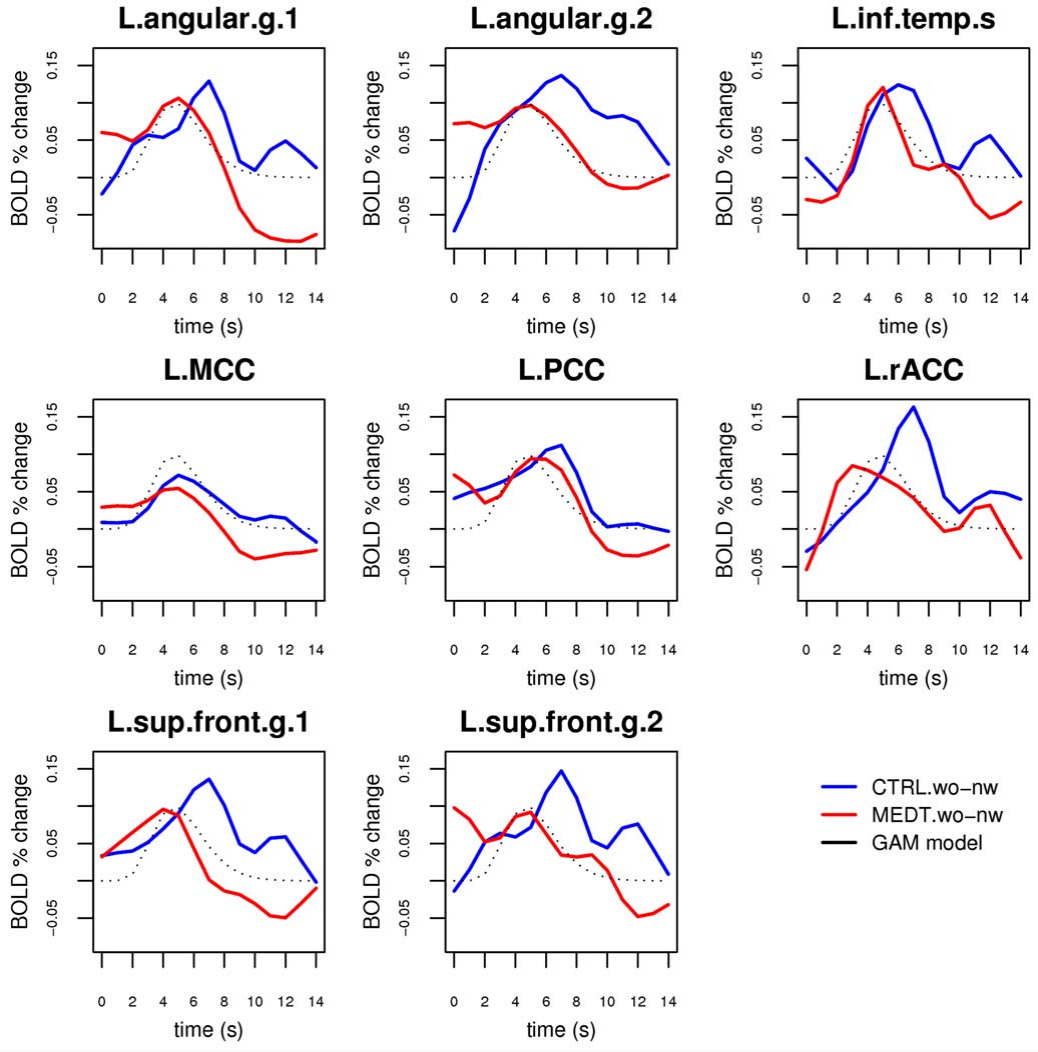
Estimates of the BOLD response associated with semantic processing in the ROI set, obtained by fitting a spline basis set model for the hemodynamic function and subtracting the average response to nonwords (“nw”) from the average response to words (“wo”) in meditators and controls. The Gamma function model for a standard hemodynamic response is plotted as a black dotted line for reference.

**Figure 4:**
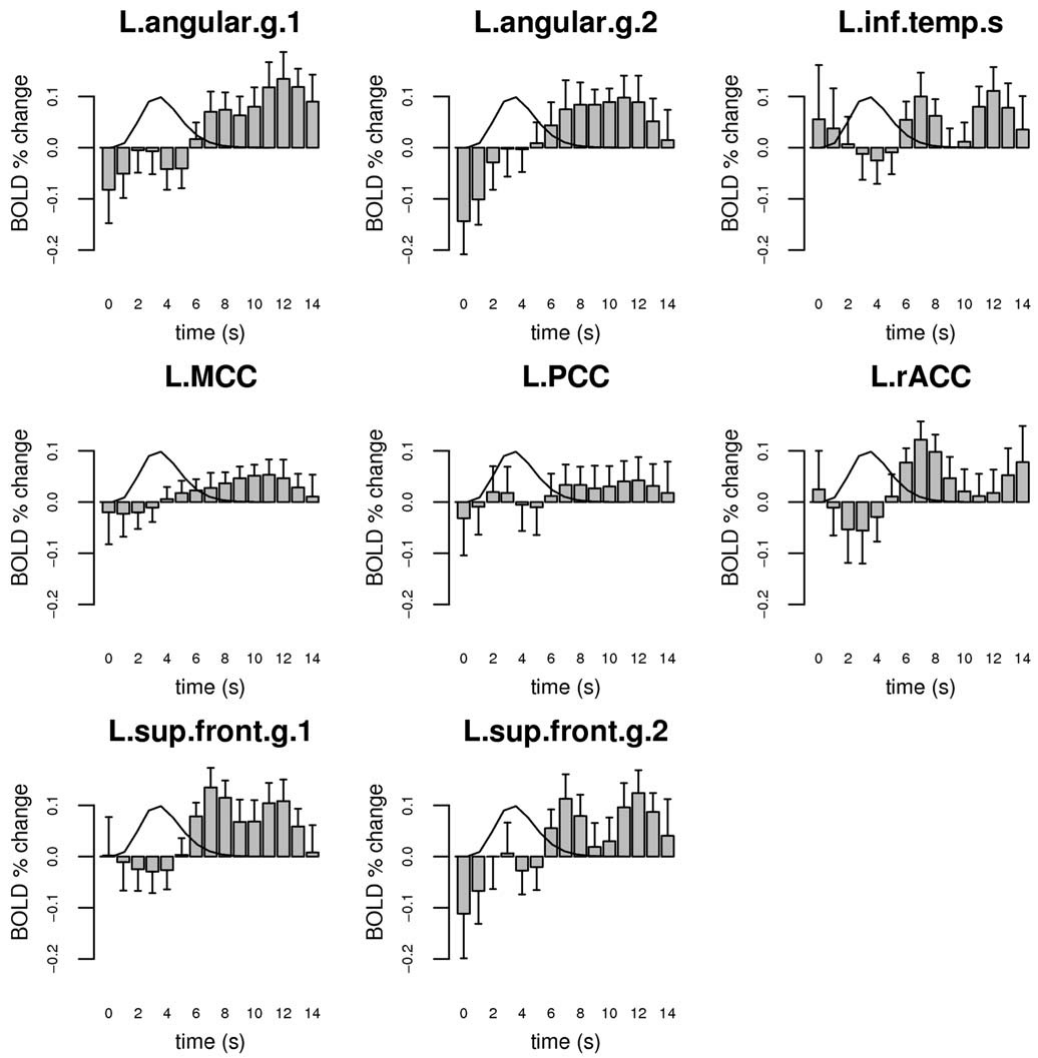
Difference between controls and meditators (CTRL-MEDT) in the estimated profile of the BOLD response related to conceptual activity. Error bars represent standard errors and a reference Gamma function model for the BOLD response to a single brief stimulus is plotted as a black line for reference.

Notably, the contrast *words-nonwords*, when the hemodynamic response was modeled as a simple Gamma function, was not significantly different between controls and meditators in any ROI (all *t*-tests, *p*>0.2). It is important to recognize, however, that this corresponds to a real lack of difference in the response properties of meditators and control only insofar as the Gamma function models the full extent of the hemodynamic time course satisfactorily. In particular, since we were interested in the residual semantic processing occurring after the subjects responded to the stimulus, we examined the ROIs' activation profile in more detail by modeling the hemodynamic time course with a more general basis set of spline functions. The estimated event-related time courses for the stimulus response component associated with conceptual processing (see Methods), obtained by subtracting the estimated waveform for nonword stimuli from the estimated waveform for word stimuli, showed a clear difference between meditators and controls in the peri-stimulus interval following the peak of the Gamma model (Figure 3). The event-related time course of this difference is plotted explicitly in Figure 4. A repeated-measure ANOVA on the cumulative measure of the BOLD activity associated with semantic processing in the 6–14 s post-stimulus period, with group as a between-subject factor and ROI as a within-subject factor (see Methods), showed significant main effects of group (CTRL>MEDT, *F*(1,22) = 12.3, *p* = 0.002) and ROI (*F*(7,154) = 3.5, *p* = 0.001), and no interaction (*F*(7,154) = 1.3, *p* = 0.2). Post-hoc tests of group differences within each ROI revealed that this effect was common across all ROIs, with the exception of the middle and posterior cingulate areas (Table 3).

**Table 3:** Tests of group differences in the cumulative measure of the BOLD signal related to semantic processing in the 6–14 s post-stimulus period within each ROI.

*Region*	*F-stats*	*p-value*
L MCC	2.26	0.13
L sup frontal g (1)	11.89	0.0007^***^
L sup frontal g (2)	8.91	0.0033^**^
L angular g (1)	12.49	0.0005^***^
L angular g (2)	8.45	0.0042^**^
L PCC	1.54	0.22
L rACC	5.93	0.016^*^
L inf temporal g	6.07	0.015^*^

The reported *p*-values are Bonferroni corrected. Significance codes: ^*^<0.05, ^**^<0.01, ^***^<0.001.

## Discussion

In this study, we employed a simple lexical decision paradigm to investigate whether the regular practice of meditation can affect the dynamics of implicit conceptual processing and, more specifically, to test whether experienced meditators would display the ability to abbreviate the duration of neural processing triggered by semantic stimuli during meditation. To this end, we first identified the brain regions associated with conceptual processing across the two groups of subjects, and then estimated the temporal course of the stimulus-evoked response in these regions. The results support the hypothesis that the regular practice of Zen meditation enhances the capacity for voluntary regulation of spontaneous mental activity. In regions of the default network, meditators displayed a BOLD response related to semantic processing that was characterized by a reduced post-stimulus tail compared to control subjects. A possible explanation for this finding is that meditators, given their practice history, had an advantage over control subjects in the experimental meditative task of re-focusing attention on the breath after having processed and responded to the presented stimuli. It is interesting to note that in a few ROIs and especially in the L.angular.g.1 (Figure 3), a key region in conceptual elaboration [27], [28], [29], the BOLD signal related to semantic processing drops to a level below baseline in the post-stimulus period in meditators. This finding may indicate that the active process of regulating the stimulus-triggered conceptual processing by re-focusing on the breath, is in meditators so effective as to bring the level of semantic processing temporarily below the level of the normal baseline. During the baseline periods, i.e. the intervals where no stimuli are presented, a certain amount of spontaneous thinking is likely to occur in both groups of subjects (although perhaps less so in meditators), but in the period immediately following a stimulus response, the strong engagement of the executive function involved in re-focusing attention on the breath may cause the level of conceptual processing to drop below the normal baseline level; this is usually the case for deactivations in the default mode network, which increase in amplitude as executive demands increase [26].

Notably, the conceptual processing evoked by the word stimuli in the lexical decision task was completely implicit, in the sense that no explicit conceptual elaboration of the stimuli was required in order to perform the task correctly. The task relied on the assumption that the visual presentation of a lexical stimulus with semantic content, insofar as it was recognized as a “real English word”, would automatically activate a cluster of semantic associations whose neural correlates could be identified by the contrast *words-nonwords*. The choice of implicit rather than explicit conceptual processing was motivated by the desire to mimic, to a certain extent, the properties of spontaneous, task-unrelated thoughts. In this sense, the snippets of semantic content delivered at random times within the baseline meditative condition were employed as “seeds” for triggering from the outside, and in an experimentally controlled fashion, the automatic activation of at least a subset of the conceptual cluster linked to the presented word.

We did not observe any difference in reaction times or errors between controls and meditators. It could have been expected that meditators would exhibit a prompter response to the stimuli, by virtue of their training in being less distracted by spontaneous thoughts. We note, however, that subjects in our study were instructed to concentrate on their breathing and that, therefore, responding to the stimulus required a switching from an internally to an externally oriented attentional modality, a process with a significant cost in terms of reaction times that may have masked such an effect. In the actual practice of *zazen*, on the other hand, a great importance is placed on a regulated sitting posture and a mental attitude of openness to the arising of perceptions without allowing one's attention to be sequestered by them. While both of these components are thought to promote a state of mental readiness that may decrease reaction times to an external stimulus, they were not included in our protocol for pragmatic reasons, *i.e.*, that the simplified meditative technique of breath concentration could be easily adopted by the non-meditators, as well as the impossibility of assuming a seated posture in the scanner. These factors may explain the observed lack of differences in the behavioral results between meditators and controls.

The pattern of activation identified by the contrast *words-nonwords* included the typical nodes of the default mode network, replicating the results obtained by Binder and colleagues [11] in a study employing the same stimuli in a fast event-related design. This is also consistent with several reports implicating regions of the default mode network in semantic processes of either task-related [10], [24], [30], [31] or task-unrelated nature [13], [23], with a complex interaction of the two with respect to memory formation [32], [33], [34]. The activated clusters were restricted to the left hemisphere, which is to be expected given the lexical nature of the task and well-known left-hemisphere dominance in language function. Notably, meditators and controls exhibited no difference for the *words-nonwords* contrast in these regions when the hemodynamic response was modeled by a simple Gamma function. A whole-brain analysis directly comparing meditators and controls for the same contrast (*words-nonwords*) also revealed no significant group differences at the statistical threshold of α<0.05, corrected (single-voxel *p*<0.001, cluster size *k*>27 voxels). It is important to note that the response model based on the convolution of the stimulus presentation sequence with a simple Gamma function was generally able to detect the initial transient rise in the signal, but could only partially fit a response that was more extended than the canonical hemodynamic response to a brief simple stimulus. Since the duration of the processing triggered by the presentation of the stimuli was a quantity of interest in our study and unknown *a priori*, we estimated the actual time course of the response in the selected set of ROIs by modeling the stimulus response with a basis set of cubic splines. Using this method, we were able to detect a decreased duration of the BOLD response related to conceptual processing in most regions identified by the initial analysis in meditators versus controls (Table 3, Figure 4). This effect was particularly prominent in the left angular gyrus and the left superior frontal gyrus, regions whose level of activity has been reported to be strongly correlated with the amount of task-independent thoughts in a recent study [23]. The only ROIs that did not display a significant group effect were the middle cingulate cortex, which is not commonly considered part of the default network, and the posterior cingulate cortex, which also displayed a lesser correlation with task-independent thoughts compared to the other regions in the above mentioned study [23]. The medial parietal cortex (including posterior cingulate, retrosplenial cortex, and precuneus) has been hypothesized to occupy a rather early stage in processing semantic information [35], which could explain why we did not detect a significant group difference in this region for the post-stimulus semantic activity. From this perspective, an effect of sustained semantic processing is more likely to be observed in areas that occupy later processing stages, in particular higher-order associative areas such as the angular gyrus and regions of the prefrontal cortex, which are optimally suited to maintain an organized pattern of activity for extended durations [36], [37]. There is in fact considerable evidence indicating that the region around the angular gyrus, corresponding roughly to Brodmann areas 39/40 and originally described by Norman Geschwind [38], is a key structure in semantic processing [28], [29] and may have had an evolutionary role in the development of language [39]. The angular gyrus has also been hypothesized to play a specific role in the default network by integrating semantic information into an ongoing context, and has recently been shown to display later-stage responses to semantic material similar to those observed here [40].

The present work contributes novel data to the burgeoning field of meditation studies in the context of modern neuroscience (see [5], [41] for reviews), as well as to the research on mind-wandering and stimulus-independent thoughts [42]. While most of the recent literature has focused on the effect of meditative practices on attentional processes [43], [44], [45], [46], [47], [48], and a substantial effort has been devoted to investigate the processes underlying mind-wandering under non-meditative conditions [13], [49], [50], [51], [52], [53], there has not yet been, to our knowledge, any previous attempt to characterize the neural correlates of conceptual processing during meditation. Importantly, this is an area of research with potential clinical relevance for psychiatric conditions characterized by excessive rumination [54], such as obsessive-compulsive disorder [55], anxiety disorder [56], and major depression [57], [58], [59].

In closing, we would like to indicate some limitations of the present study that should be explored by further work. First, the employed cross-sectional experimental design cannot rule out the possibility of a selection bias where the observed effect is not due to the difference in meditative experience between the two groups but to some pre-existing hidden variable; a longitudinal design, with random assignment of subjects to a meditation training group and a group with a control intervention, would be able to detect differences due to training with greater confidence, albeit at the likely price of investigating effects limited to short-term training. Secondly, the study was not designed to assess behavioral correlates of the fMRI finding of a faster post-stimulus renormalization, in meditators compared to controls, of the BOLD signal related to semantic processing; future work should explore the use of behavioral probes that can directly assess the level of conceptual processing in the post-stimulus period without critically interfering with the main paradigm. Thirdly, while the study was sufficiently powered to detect the reported effects, it could clearly benefit from a larger sample size. Finally, for the sake of ecological validity and in view of potential clinical applications, the adopted experimental paradigm could be expanded to include stimuli with strong emotional content [58] and organized in full sentences with richer semantic structure [60].

## Methods

### Subjects

Twelve Zen meditators (MEDT) with more than 3 years of daily practice were recruited from the local community and meditation centers, along with 12 control subjects (CTRL) who had never practiced meditation. The groups were matched by sex (MEDT = 10 M, CTRL = 9 M), age (mean±SD: MEDT, 37.3±7.2 years; CTRL, 35.3±5.9 years; two-tailed two-sample *t*-test: *p* = 0.45), and education level (mean±SD: MEDT, 17.8±2.5 years; CTRL, 17.6±1.6 years; *p* = 0.85). All participants were native speakers of English and right-handed, except one meditator who was ambidextrous. Subjects gave written informed consent for a protocol approved by the Emory University Institutional Review Board.

### Experimental task

Subjects of both groups were instructed to pay attention to their breathing throughout the fMRI scan and return to it every time they found themselves distracted by thoughts, memories, or sensations; a fixation cross was kept on the MRI display screen to help concentration and avoid excessive eye movement. The choice of having both meditators and controls engage in a simplified meditative condition, as opposed to having the controls simply “rest” and the meditators meditate, was motivated by the desire to equalize procedurally the two experimental groups as much as possible, so that any observed group difference in brain activation would be more easily attributable to a difference in meditative experience and training. It was explained to the subjects that this “meditative” baseline condition would be interrupted at random times by the appearance of a string of letters on the screen: they should indicate with their left hand, by pressing either the index or the middle finger button of a response box, whether the stimulus was a “real English word” (index finger) or not (middle finger) and promptly return their attention to their breathing. The lexical decision task was adapted from Binder *et al.* [11] and employed a subset of the same phonologically and orthographically matched words and nonwords (50 items each). Using the routine *RSFgen* in the software package AFNI [61], the temporal schedule of the stimuli was selected as the one with the greatest statistical efficiency from a Monte Carlo simulation of 10,000 randomly generated stimulus sequences. The onset times of half of the stimuli in each category were subsequently jittered by an interval of TR/2 s in order to improve the statistical estimation of the hemodynamic response function. Given the length of the imaging run (≈20 min), the resulting schedule was sparse enough to allow a reasonable establishment of the baseline condition of refocusing attention to the breathing (inter-stimulus interval: range 1.4–72.9 s, median = 8.2 s, IQR = 12.9 s). Stimulus presentation and response collection were implemented using the Cogent 2000 software package (Wellcome Department of Imaging Neuroscience, http://www.fil.ion.ucl.ac.uk/Cogent2000.html).

### MRI acquisition and preprocessing

Scanning was performed with a 3.0 Tesla Siemens Magnetom Trio scanner. The imaging session consisted of the acquisition of a T1-weighted high-resolution anatomical image (MPRAGE, 176 sagittal slices, voxel size: 1×1×1 mm), followed by the acquisition of a single series of functional images (gradient-echo echo-planar, 520 scans, 35 axial slices, voxel size: 3×3×3 mm, TR = 2.35 s, TE = 30). During the acquisition of the anatomical image, subjects practiced a shorter version of the lexical decision task with word and nonword items from a different set (50 items for each category, inter-stimulus interval = 3 s). During the functional scan, subjects engaged in the simplified meditative condition and phasically responded to the lexical decision task as described in the previous section.

For each subject, the functional scans were corrected for the slice acquisition timing schedule and head movement; the T1-weighted anatomical image was spatially registered with a 6-parameter rigid-body transformation to the average of the motion-corrected functional images and subsequently warped to the Montreal Neurological Institute brain template using a 12-parameter affine transformation followed by non-linear deformations; the estimated warping parameters were applied to the functional scans, which were then spatially smoothed with an 8 mm full-width-half-maximum (FWHM) Gaussian isotropic kernel. Image processing was performed with the freely available software packages AFNI (http://afni.nimh.nih.gov) and SPM5 (http://fil.ion.ucl.ac.uk/spm/software/spm5).

### Statistical analysis

Statistical analyses on behavioral data and quantities derived from the estimation of fMRI response parameters were performed using the freely available software package R (http://www.r-project.org). All statistical tests were two-sided, unless specified otherwise.

### Behavioral data

The response times from the lexical decision task (Table 1) were submitted to a repeated-measure ANOVA, with group (CTRL, MEDT) as a between-subject factor and stimulus type (word, nonword) as a within-subject factor.

### Imaging data

A general linear model (GLM) was fitted to the fMRI time series for each subject. The GLM included two regressors representing the expected fMRI response to word and nonword stimuli, obtained by convolving the stimuli temporal sequence with a Gamma function model of the hemodynamic response [62], an additional regressor modeling the response to error trials, the six motion parameters estimated during the head movement correction phase of the preprocessing, and a basis set of 10 functions representing a Legendre polynomial of the 9*^th^* order, modeling low-frequency confounds. The spatial images encoding the parameter estimates (“betas”) for word and nonword regressors were then individually scaled to represent a voxel-wise percent signal change with respect to each voxel's temporal mean.

In order to localize the brain regions involved in conceptual processing across the two groups, the data from meditators and controls were pooled together and a random-effects model was implemented as a paired two-sample *t*-test on the beta images corresponding to the effects of words and nonwords. The resulting statistical *t*-map was thresholded at the combined single-voxel significance level of *p*<0.001 with cluster size *k*>27 voxels. These values were determined by a Monte Carlo simulation of the cluster size distribution under the null hypothesis [63] to yield a family-wise error rate of α<0.05.

The clusters identified in this analysis served as regions of interest (ROIs) for a more detailed investigation of the amplitude of the response to words and nonwords, as well as for the estimation of the temporal dynamics of conceptual processing in the two groups of subjects. To this purpose, a new GLM was fitted to each subject's fMRI data, where the hemodynamic response to words and nonwords was now modeled with a basis set of seven cubic splines spaced one TR (2.35 s) apart and spanning the interval from 0 to 14.1 s post-stimulus. The set of fitted splines was then resampled at a 1 s temporal resolution, to give a reconstructed event-related response on a 1 s temporal grid, and averaged within each ROI.

It is important to note that, in our task, responses to words were composed of at least two components: (1) a generic “circuit-breaker” component also present for nonword stimuli and associated with the momentary interruption of the meditative task, the processing of an external visual stimulus, and the motor response; and (2) a conceptual component related to the automatic cascade of semantic associations generated by the presented word, which was absent for nonword stimuli. In order to obtain an estimate of the time course of the latter component, more specifically linked to conceptual processing, the event-related response relative to nonwords was subtracted from the response relative to words, for each subject and each ROI. Finally, to test the original hypothesis of a reduced “semantic reverberation” in meditators compared to controls following the initial processing of the stimulus, the values of the time course representing the conceptual component were summed across all the time points following the peak of the “canonical” hemodynamic response represented by the Gamma function (6–14 s post-stimulus), and the resulting sums were entered into a repeated-measure ANOVA with group (CTRL, MEDT) as a between-subject factor and ROI as a within-subject factor. Post-hoc tests of group differences within each ROI were performed and Bonferroni correction was applied to adjust for multiple comparisons.
